# The Prognostic, Predictive and Clinicopathological Implications of KRT81/HNF1A- and GATA6-Based Transcriptional Subtyping in Pancreatic Cancer

**DOI:** 10.3390/biom15030426

**Published:** 2025-03-17

**Authors:** Michael Guenther, Sai Agash Surendran, Lea Margareta Steinke, Iduna Liou, Melanie Alexandra Palm, Volker Heinemann, Michael Haas, Stefan Boeck, Steffen Ormanns

**Affiliations:** 1 Innpath Institute of Pathology, Tirol Kliniken, 6020 Innsbruck, Austria; michael.guenther@innpath.at; 2 Institute of Pathology, Faculty of Medicine, Ludwig-Maximilians-University, 80337 Munich, Germany; 3 Department of Hematology and Oncology, München Klinik Neuperlach, 81737 Munich, Germany; volker.heinemann@med.uni-muenchen.de (V.H.); michael.haas@muenchen-klinik.de (M.H.); stefan.boeck@muenchen-klinik.de (S.B.); 4 Department of Internal Medicine III, Grosshadern University Hospital, Ludwig-Maximilians-University, 81377 Munich, Germany; 5 Institute of Pathology, Neuropathology and Molecular Pathology, Medical University Innsbruck, 6020 Innsbruck, Austria

**Keywords:** pancreatic cancer, palliative chemotherapy, adjuvant chemotherapy, transcriptional subtyping

## Abstract

Background: Transcriptional subtypes of pancreatic ductal adenocarcinoma (PDAC) based on the expression of hallmark genes may have prognostic implications and potential predictive functions. The two most employed subtyping markers assess the combined expression of KRT81 and HNF1A or of GATA6 alone, which can be detected by immunohistochemistry (IHC). This study aimed to determine the prognostic or predictive impact of both subtyping marker panels in two large cohorts of advanced and resected pancreatic ductal adenocarcinoma (PDAC) patients. Methods: Transcriptional subtypes were determined by combining the expression of KRT81/HNF1A or assessing GATA6 expression alone by IHC in samples of two independent PDAC patient cohorts (advanced PDAC *n* = 139 and resected PDAC *n* = 411) as well as in 57 matched primary tumors and their corresponding metastases. RNAseq-based expression data of 316 resected PDAC patients was analyzed for validation. Results: Transcriptional subtypes widely overlapped in both marker panels (χ^2^ *p* < 0.001) but switched during disease progression in up to 31.6% of patients. They had a modest impact on the patients’ prognosis in both cohorts, with longer overall survival (OS) for patients with KRT81−/HNF1A+ or GATA6+ tumors but better progression-free survival (PFS) and disease-free survival (DFS) in patients with KRT81+/GATA6− tumors treated with palliative or adjuvant gemcitabine-based chemotherapy. RNAseq expression data confirmed the findings. Conclusions: Transcriptional subtypes have differential responses to palliative and adjuvant gemcitabine-based chemotherapy and may change during disease progression. Both employed subtyping marker panels are equivalent and may be used to inform clinical therapy decisions.

## 1. Background

Although modest improvements in the prognosis of pancreatic ductal adenocarcinoma (PDAC) patients were achieved in recent years, mainly through innovative randomized controlled trials (RCT) on novel chemotherapy (CTX) regimens, the survival probability of the average PDAC patient remains desperately low [1], even for patients in early disease stages [2]. For the minority of patients diagnosed with resectable disease, adjuvant therapy has become the standard of care, with the active but toxic regimen FOLFIRINOX as first choice for the clinically fit patient and less active but less toxic gemcitabine-based regimens for the less fit [3]. Large sequencing studies have revealed the molecular background of the disease on the genetic as well as the transcriptional level, which resulted in the identification of transcriptional subtypes based on the expression of specific hallmark genes [4]. Thus, several transcriptional subtyping systems were proposed, each of which showed a differential prognostic impact of the subtypes identified [4]. Some studies even proposed prognostic implications of transcriptional subtyping, which largely rely on complex, RNA-based methodologies such as RNA-sequencing (RNAseq), which are hard to establish in routine diagnostics. Therefore, efforts were made to break down complex subtyping systems to simpler approaches applicable in routine clinical practice by detecting the expression of each subtype’s hallmark genes through immunohistochemistry (IHC) [5,6]. Although these approaches resulted in valuable information on the potential clinical implications of the subtypes identified, their meaning to inform clinical decision-making was modest at best [6,7]. Moreover, most studies did not examine the clinical significance of transcriptional subtypes with respect to clinicopathological parameters such as disease stage or the therapies applied, specifically in the adjuvant setting, and no study to date compared the different subtyping systems with each other in the same set of samples. Thus, in the present study, we examined the prognostic and potentially predictive impact of the two most employed IHC-based subtyping marker panels, one based on the expression of KRT81/HNF1A and the other based on the expression of GATA6. We applied both subtyping marker panels in large cohorts of advanced PDAC and resected PDAC patients, compared them with each other and determined how the metastatic process might affect the predominant subtype.

## 2. Materials and Methods

### 2.1. Study Outline and Patient Selection

Two cohorts were examined for the present study. The advanced pancreatic cancer (aPDAC) cohort consisted of n = 139 patients with aPDAC treated with conventional gemcitabine or non-gemcitabine-containing systemic chemotherapy within clinical trials [8,9]. The resected pancreatic cancer (rPDAC) cohort consisted of n = 411 patients resected for PDAC at a single academic center between 2000 and 2016 receiving either no or a non-gemcitabine-based adjuvant therapy, or a gemcitabine-based adjuvant chemotherapy after resection. For both cohorts, only patients older than 18 years of age with histologically confirmed pancreatic ductal adenocarcinoma were included. In the rPDAC cohort, patients deceased from perioperative complications within 30 days after surgery were excluded. The use of both cohorts for translational research purposes was described previously [10]. Archival formalin-fixed paraffin embedded (FFPE) histologically confirmed tumor tissue of primary tumors and metastatic tissue was collected from the pathology laboratories where the diagnosis of PDAC was first established. Patients’ overall survival (OS), disease free survival (DFS) and progression free survival (PFS) were calculated as previously described [10]. Written informed consent for the use of tumor material and clinical data was obtained from advanced PDAC patients upon study enrollment or before palliative chemotherapy initiation. The ethics committee of the LMU medical faculty approved the use of patient material and data in the resected PDAC cohort (project 20-081).

### 2.2. Tumor Samples and Immunohistochemistry

Tissue microarray (TMA) construction was performed as previously described [10]. GATA6 expression was detected on 4-µm-thick sections by immunohistochemistry using anti-GATA6 polyclonal rabbit antibody (PA1-104, Thermo Fisher, Germering, Germany) at 1:200 dilution. Immunohistochemical detection of KRT81 and HNF1A was performed as described previously [11]. Appropriate positive controls were included in each staining run (human normal tonsil for KRT81, duodenal mucosa for HNF1A and normal exocrine pancreas for GATA6, Supplementary Figure S1A–C). The expression pattern and expression strength were independently evaluated by two pathologists (M.G., S.O.) blinded to the patient outcome and discrepant cases were discussed until agreement was reached. Tumors were classified as follows: samples with ≥30% KRT81- or HNF1A-positive tumor cells were considered positive for each marker. For GATA6 expression, tumors with distinct nuclear staining were considered GATA6 positive. Microphotographs were acquired as described previously [10].

### 2.3. Statistical and In Silico Analyses

Kaplan–Meier curves, Cox regression analyses and cross tabulations were computed using SPSS software (version 29, IBM, Ehningen, Germany), considering a *p*-value of ≤0.05 as statistically significant. Propensity score matching, as well as analysis of publicly available gene expression data and the patients’ corresponding clinical data, was carried out as described before [10]. RNA sequencing data and clinical information of the ICGC-CA cohort were downloaded from the International Cancer Genome Consortium (ICGC) data portal [12]. Optimal cut points defining low and high expression of KRT81/HNF1A or GATA6 were determined using maximally selected rank statistics (R package: MaxStat v. 0.7-25). Genomic analysis and data visualization were conducted using cBioPortal for Cancer Genomics [13].

## 3. Results

### 3.1. Transcriptional Subtypes According to the Expression of KRT81/HNF1A or GATA6 Widely Overlap but May Change During Metastatic Progression

**Figure 1:**
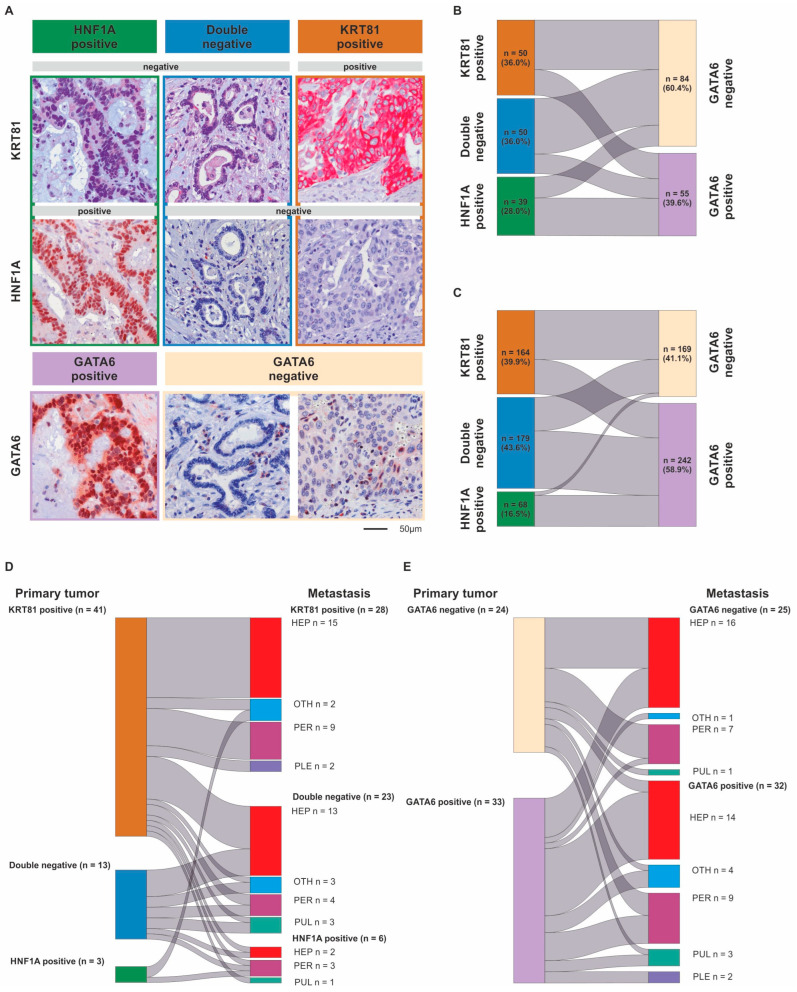
Differential expression of KRT81, HNF1A and GATA6 in pancreatic cancer. Immunohistochemical detection of *KRT81*, *HNF1A and GATA6* expression in exemplary PDAC samples at 200-fold magnification. Scale bar indicates 50 µm (**A**). Comparison of tumors’ transcriptional subtypes based on the expression of KRT81/HNF1A or GATA6 in advanced (**B**) and resected PDAC (**C**) as well as between the primary tumor and its corresponding metastasis using subtyping based on KRT81/HNF1A expression (**D**) or GATA6 expression (**E**).

Of the 139 samples in the advanced PDAC (aPDAC) cohort, 36.0% (n = 50) were KRT81+, 36.0% (n = 50) were double negative and 28.0% (n = 39) were HNF1A+, whereas 60.4% (n = 84) were GATA6− and 39.6% (n = 55) were GATA6+. In the resected PDAC (rPDAC) cohort, 39.9% (n = 164) were KRT81+, 43.6% (n = 179) were double negative and 16.5% (n = 68) were HNF1A+, whereas 41.1% (n = 169) were GATA6+ and 58.9% (n = 242) were GATA6− (Figure 1A). Tumors displaying expression of both KRT81 and HNF1A (double positive), which was the case in 16.5% of the samples in the aPDAC cohort and 7.1% in the rPDAC cohort, were categorized according to the marker showing a predominant expression pattern. This approach was backed by highly similar prognostic implications of the subsequently assessed subtypes in the double positive cases compared to the single positive ones (Supplementary Table S1). Both subtyping marker panels overlapped widely in both cohorts (χ^2^ *p* < 0.001 each), with the HNF1A+ subtype being mostly GATA6+ (Supplementary Table S2). However, a significant proportion of KRT81+ samples displayed GATA6 expression in both cohorts and no clear trend towards a GATA6-based subtype was observed for the double negative samples, which were rather GATA6− in the aPDAC cohort and rather GATA6+ in the rPDAC cohort (Figure 1B,C). For both panels, the assessment of each tumor subtype remained remarkably stable when several tumor tissue punches from different FFPE blocks were compared separately (Supplementary Table S3).

To assess how metastatic progression might affect the tumor subtype, we examined to which extent the primary tumor and metastatic tissue subtypes were interrelated in metastases synchronous and metachronous to their corresponding primary in 57 cases. There was a strong correlation between the primaries’ and the metastases’ subtypes (*p* = 0.001, Figure 1D,E, Supplementary Table S6), but we observed a subtype switch according to KRT81/HNF1A expression in 18 cases (31.6%). This was not associated with synchronous or metachronous metastasis (Supplementary Table S4) but came with a highly significant trend towards a prognostically more favorable subtype (Supplementary Table S5). More than half (n = 28) of the n = 41 KRT81+ cases remained KRT81+ in their corresponding metastasis; the rest were mostly double negative and only three cases switched to HNF1A+. Double negative cases mostly remained double negative during metastasis, whereas the few (n = 3) HNF1A+ cases were split between KRT81 positivity and HNF1A positivity in their metastatic tissue (Figure 1D, Supplementary Table S6). In contrast, transcriptional subtype according to GATA6 expression remained stable in most cases with 9 of 34 cases switching from GATA6– to GATA6+ and 7 of 23 cases switching from GATA6+ to GATA6– (Figure 1E, Supplementary Table S6). Here, subtype switching occurred more frequently in synchronous metastases (Supplementary Table S4). Neither the primary tumor’s or metastases’ subtypes nor the occurrence of a subtype switch was associated with metastasis localization (Supplementary Table S7).

### 3.2. Transcriptional Subtypes Affect Patient Outcome Dependent on Palliative Therapy in Advanced PDAC Patients

**Figure 2:**
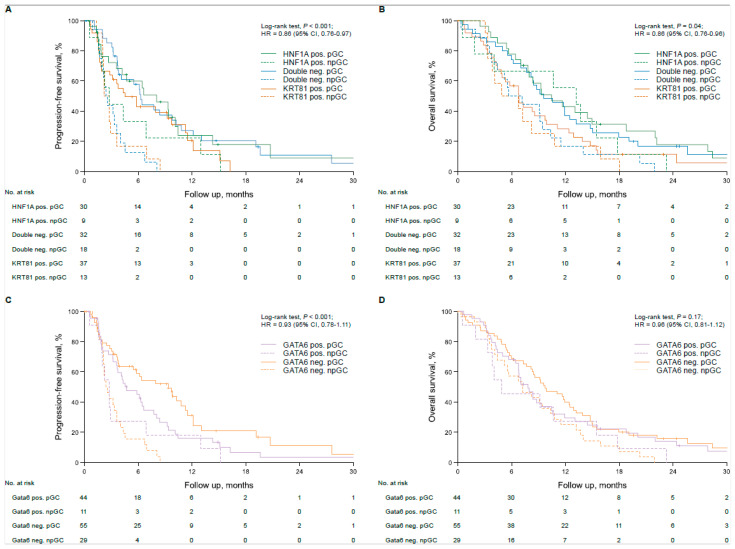
Transcriptional subtypes are associated with therapy response in first-line gemcitabine-treated advanced pancreatic cancer patients. Univariate analyses (Kaplan–Meier curves and log-rank tests) for PFS and OS in the subtypes based on KRT81/HNF1A expression stratified by first-line chemotherapy (**A**,**B**) and in the subtypes based on GATA6 expression stratified by first-line chemotherapy (**C**,**D**). Crossed lines indicate censored cases.

The baseline characteristics, outcome and clinicopathological variables of the aPDAC patient cohort were previously described [10]. In the unstratified cohort, transcriptional subtypes according to KRT81/HNF1A expression implied a statistically non-significant trend for patient OS, with HNF1A+ cases having the best, double negative cases an intermediate and KRT81+ the worst outcome (OS 6.8 vs. 9.1 vs. 10.7 months, *p* = 0.08, HR = 1.27 95%, CI 1.02–1.59, Supplementary Figure S2A), which was not reflected in PFS times (PFS 3.6 vs. 4.1 vs. 6.4 months, *p* = 0.42, HR = 1.16 95% CI 0.91–1.48, Supplementary Figure S2B). Similarly, subtyping based on GATA6 expression had no prognostic impact in the unstratified patient cohort (PFS 4.2 vs. 4.3 months, *p* = 0.43, HR 1.16 95%CI 0.80–1.69; OS 7.8 vs. 9.2 months, *p* = 0.76, HR 1.06, 95% CI 0.74–1.51, Supplementary Figure S2C,D). Only KRT81/HNF1A-based subtypes showed a trend towards statistical significance in multivariate analyses (*p* = 0.07, Supplementary Table S8) and transcriptional subtypes were not associated with the patients’ clinicopathological variables (Table 1). Stratification of patient subgroups according to the applied type of first-line palliative treatment revealed a significant impact of tumor transcriptional subtype on patient outcome, as patients with double negative tumors derived the most benefit from palliative gemcitabine-based chemotherapy (pGC) compared to palliative non-gemcitabine-based chemotherapy (pnGC, PFS 6.3 vs. 2.4 months, *p* < 0.001, HR 0.26, 95%CI 0.13–0.52; OS 9.6 vs. 5.7 months, *p* = 0.04, HR 0.53, 95%CI 0.29–0.97, Figure 2A,B). Similarly, patients with KRT81+ tumors showed favorable PFS times with pGC compared to pnGC (PFS 4.5 vs. 2.2 months, *p* = 0.02, HR 0.43, 95%CI 0.21–0.90 Figure 2A), but no significant impact of palliative chemotherapy on OS times (OS 6.8 vs. 4.7 months, *p* = 0.47, HR 0.78 95%CI 0.40–1.52, Figure 2B). Interestingly, the type of palliative chemotherapy had no significant prognostic impact in patients with HNF1A+ tumors (pnGC vs. pGC, PFS 6.6 vs. 2.7 months, *p* = 0.28, HR 0.65 95%CI 0.30–1.42; OS 13.2 vs. 9.3 months, *p* = 0.60, HR 0.82, 95%CI 0.38–1.75 Figure 2A,B), which was also confirmed in multivariate analyses (Supplementary Table S9). Applying the GATA6-based subtypes paralleled these findings, as patients with GATA6− tumors benefited from pGC compared to pnGC, whereas no significant prognostic impact of the type of palliative chemotherapy was detected for patients with GATA6+ tumors (GATA6− PFS 9.3 vs. 2.4 months, *p* < 0.001, HR 0.27, 95%CI 0.15–0.48; OS 9.7 vs. 7.2 months, *p* = 0.04, HR 0.60, 95%CI 0.38–0.97; GATA6+ 5.0 vs. 2.7 months, *p* = 0.21, HR 0.65 95%CI 0.33–1.29; OS 7.8 vs. 4.9 months, *p* = 0.34, HR 0.72 95%CI 0.37–1.42, Figure 2C,D), which was confirmed for PFS in multivariate analyses (Supplementary Table S9).

### 3.3. Transcriptional Subtypes Affect Patient Outcome Dependent on Adjuvant Therapy in Resected PDAC Patients

**Figure 3:**
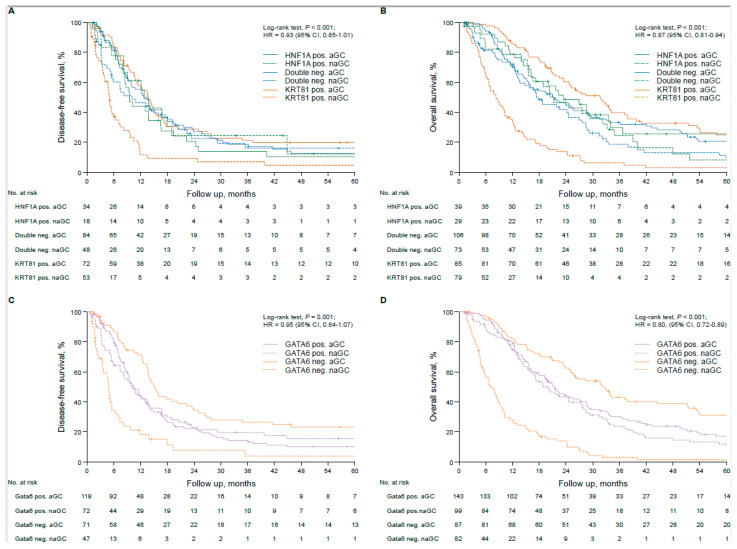
Transcriptional subtypes are associated with therapy response in resected pancreatic cancer patients treated with adjuvant gemcitabine-based chemotherapy. Univariate analyses (Kaplan–Meier curves and log-rank tests) for DFS and OS in the subtypes based on KRT81/HNF1A expression stratified by gemcitabine-based adjuvant treatment (**A**,**B**) and in the subtypes based on GATA6 expression stratified by gemcitabine-based adjuvant treatment (**C**,**D**). Crossed lines indicate censored cases.

In the unstratified rPDAC cohort, the tumor subtype had statistically non-significant implications for the patients’ prognosis, with KRT81 positivity conferring the worst, double negative an intermediate and HNF1A positivity the best outcome (OS 17.1 vs. 19.3 vs. 22.3 months, *p* = 0.34, HR 0.87, 95% CI 0.81–0.94; DFS 9.7 vs. 12.1 vs. 12.4 months, *p* = 0.47, HR 1.08, 95%CI: 0.90–1.28, Supplementary Figure S3A,B), which turned out to be an independent prognostic marker for OS in multivariate analyses (Supplementary Table S10). Similarly, GATA6 expression was associated with a statistically insignificant trend towards better prognosis (OS 20.7 vs. 15.2 months, *p* = 0.20, HR 0.87, 95% CI 0.69–1.08, Supplementary Figure S3C) but showed no tendency for differences in DFS (Supplementary Figure S3D), although it turned out to be an independent prognosticator for OS in multivariate analyses (*p* = 0.006, HR 0.73, 95%CI 0.58–0.91, Supplementary Table S10). Transcriptional subtypes were not associated with the patients’ clinicopathological parameters (Table 2). To test the implications of the tumor subtypes by outcome according to adjuvant chemotherapy, we calculated DFS and OS times in each subtype according to the application of adjuvant gemcitabine-based chemotherapy (aGC) compared to no or non-gemcitabine-based adjuvant chemotherapy (naGC). Interestingly, the prognostically worst KRT81+ subtype had the strongest prognostic impact on adjuvant gemcitabine-based chemotherapy (DFS 5.0 vs. 13.7 months, *p* < 0.001, HR 0.37, 95% CI 0.25–0.55; OS 8.3 vs. 31.6 months, *p* < 0.001, HR 0.26 95%CI 0.18–0.37), whereas this was not the case for the double negative subtype (DFS 10.1 vs. 13.5 months, *p* = 0.52, HR 0.88, 95%CI 0.59–1.32; OS 17.7 vs. 21.0 months, *p* = 0.08, HR 0.76, 95%CI 0.54–1.07) or the HNF1A+ subtype (DFS 12.8 vs. 9.5 months, *p* = 0.39, HR 1.32, 95%CI 0.70–2.49; OS 20.4 vs. 23.4 months, *p* = 0.38, HR 0.78, 95%CI 0.45–1.36, Figure 3A,B). Similar results were obtained for GATA6-based subtyping, where the GATA6− subtype benefitted significantly from adjuvant gemcitabine-based chemotherapy (DFS 4.8 vs. 15.1 months, *p* < 0.001, HR 0.30, 95%CI 0.19–0.46; OS 7.0 vs. 32.2 months, *p* < 0.001, HR 0.21, 95%CI 0.66–1.18), whereas it was not beneficial in the GATA6+ subtype (DFS 10.5 vs. 10.0 months, *p* = 0.62, HR 1.09, 95%CI 0.78–1.51; OS 18.9 vs. 21.5 months, *p* = 0.42, HR 0.87, 95%CI 0.66–1.18, Figure 3C,D). These findings were also reflected in 5-year survival rates (Supplementary Table S11). We confirmed these findings in multivariate analyses, which also demonstrated the prognostic impact of the patients’ clinicopathological characteristics in each subtype. Interestingly, R status was most important for DFS and OS in the KRT81+ and GATA6− subtypes, whereas in the HNF1A+ and the GATA6+ subtypes pN stage had the strongest prognostic influence (Supplementary Table S11).

### 3.4. In Silico Validation and Potential Mechanistic Background

We validated these findings in two independent publicly available PDAC cohorts (n = 138 and n = 178) with corresponding RNAseq-based gene expression data. In both datasets, just as in our cohort, patients with KRT81+ or GATA6− tumors derived the most benefit from gemcitabine-based adjuvant chemotherapy, whereas in the other subtypes the impact of aGC was minor (Supplementary Figure S4).

To explore a potential mechanistic background for the observed gemcitabine resistance in the patients with HNF1A+/GATA6+ tumors, we tested the association of the expression of HNF1A, KRT81 and GATA6 with the expression of gemcitabine-resistance-associated genes in the same RNAseq-based gene expression datasets. In both cohorts, there was a strong positive correlation with HNF1A and GATA6 expression and an inverse correlation for KRT81 with the expression of *ABCC3* and *MVP*, both known to cause gemcitabine resistance in vitro and in vivo [14,15] (Supplementary Figure S5). Of note, after adjusting for multiple testing, transcriptional subtypes did not correlate with specific molecular alterations on the genomic level (Supplementary Figure S6).

## 4. Discussion

Transcriptional subtyping in pancreatic cancer using different systems of hallmark genes and detection techniques and its potential clinical or biological impact has been reported several times to date [5,6,7,11,16,17,18,19,20,21,22,23,24,25,26] (Table S13). There is a certain consensus that there are at least two subtypes, mostly termed “basal” or “quasi-mesenchymal” and “classical” [27]. Whereas the first one is often associated with poorer outcome and sometimes higher tumor grade, exemplified by the morphological adenosquamous subtype of PDAC, the latter is associated with better prognosis and better therapy response to FOLFIRINOX (FFX) [16,28]. Other previously proposed transcriptional subtypes, such as the so called “immunogenic” or “ADEX” subtype by Bailey et al. [29], could not be entirely reproduced by other groups [30]. Thus, their existence remains a matter of debate to date. None of the previously published studies on transcriptional subtyping in PDAC examined their predictive impact on currently still widely employed therapies. Moreover, none of the IHC-based subtyping marker panels have yet been compared in the same set of patient samples and a potential subtype switch during metastatic progression—which eventually occurs in the majority of PDAC patients—has not been examined either. In this study, we employed and compared the two most widespread subtyping markers based on the robust immunohistochemical detection of KRT81 in combination with HNF1A or the detection of GATA6 alone. We show that both marker panels overlap widely and can be employed equally, which offers a cost-efficient approach to personalized cancer therapy, as specific subtypes respond differentially to therapy. For instance, patients with KRT81+/GATA6− tumors benefit more from gemcitabine-based chemotherapy in both the palliative and adjuvant settings compared to their KRT81−/GATA+ counterparts in our cohorts, which is backed by previously published data [6,31]. Thus, transcriptional subtyping by IHC could be used to inform therapy decisions in the routine clinical setting, even in cases with scarce or low-quality tumor material in which RNA-based approaches tend to fail. Our approach to categorize according to the most dominant subtype in KRT81/HNF1A-based subtyping resolved the issue of double positive cases, which were excluded from analysis in previously published studies [5,7,24,25]. Our novel finding that transcriptional subtypes may switch during metastatic progression may explain the differential therapy responses of primary tumors and metastases and implies a rationale to re-biopsy metastatic tissue to inform a subtype-based therapy choice. We also show that the R status has a much stronger prognostic impact in KRT81+/GATA6− tumors. Thus, in this subtype, it is much more important to achieve a wide R0 resection compared to others, which should be considered during resection and may justify a more aggressive surgical approach.

Its retrospective nature and the fact that no patients treated with more active regimens like FFX were included in the analyses are limitations of our study. However, not only in the adjuvant setting but also in advanced disease, most patients are still treated with gemcitabine-based chemotherapy regimens to date, as co-morbidities and frailty preclude the application of potentially more efficient but more toxic regimens like FFX [32], which in real world data do not necessarily prove to be superior to gemcitabine-based regimens [33,34].

## 5. Conclusions

In the present study we propose a simple and robust IHC-based diagnostic tool that could be implemented in therapeutic decision making in routine clinical practice. Physicians should be aware of a potential subtype switch during disease progression, which justifies a re-biopsy of metastatic lesions.

## Figures and Tables

**Table 1:** Clinicopathological patient characteristics according to KRT81/HNF1A expression and GATA6 expression in the aPDAC cohort.

	Subtype, no (%)				Subtype, no (%)		
	KRT81 Pos. (n = 50)	Double Neg. (n = 50)	HNF1A Pos. (n = 39)	*p*-Value (χ2-Test)	GATA6 Neg. (n = 84)	GATA6 Pos. (n = 55)	*p*-Value (χ2-Test)
sex							
female	30 (60.0)	29 (58.0)	20 (51.3)	0.70	38 (45.2)	22 (40.0)	0.54
male	20 (40.0)	21 (42.0)	19 (48.7)		46 (54.8)	33 (60.0)	
age group							
≤60	20 (40.0)	19 (38.0)	18 (46.2)	0.73	33 (39.3)	24 (43.6)	0.61
>60	30 (60.0)	31 (62.0)	21 (53.8)		51 (60.7)	31 (56.4)	
treatment arm							
non-gemcitabine based	13 (26.0)	18 (36.0)	9 (23.1)	0.35	29 (34.5)	11 (20.0)	0.06
gemcitabine based	37 (74.0)	32 (64.0)	30 (76.9)		55 (65.5)	44 (80.0)	
KPS							
≤80	18 (36.0)	19 (38.8)	14 (35..9)	0.95	32 (38.1)	19 (35.2)	0.73
>80	32 (64.0)	30 (61.2)	25 (64.1)		52 (61.9)	35 (64.8)	
grade group							
G1-G2	17 (34.0)	23 (46.0)	21 (53.8)	0.16	33 (39.3)	28 (50.9)	0.18
G3-G4	33 (66.0)	27 (54.0)	18 (46.2)		51 (60.7)	27 (49.1)	
stage at therapy start							
locally advanced	9 (18.0)	8 (16.0)	7 (17.9)	0.96	15 (17.9)	9 (16.4)	0.82
metastatic	41 (82.0)	42 (84.0)	32 (82.1)		69 (82.1)	46 (83.6)	

**Table 2:** Clinicopathological patient characteristics according to KRT81/HNF1A expression and GATA6 expression in the rPDAC cohort.

	Subtype, no (%)				Subtype, no (%)		
	KRT81-Positive (n = 164)	Double-Negative (n = 179)	HNF1A-Positive (n = 68)	*p*-Value (χ2 )	GATA6 Neg. (n = 169)	GATA6 Pos. (n = 242)	*p*-Value (χ2 )
sex							
female	72 (43.9)	99 (55.3)	29 (42.6)	0.06	72 (42.6)	128 (52.9)	0.04
male	92 (56.1)	80 (44.7)	39 (57.4)		97 (57.4)	114 (47.1)	
age group							
≤68	91 (55.5)	93 (52.0)	29 (42.6)	0.20	73 (43.2)	125 (51.7)	0.09
>68	73 (44.5)	86 (48.0)	39 (57.4)		96 (56.8)	117 (48.3)	
treatment arm, no (%)							
non-gemcitabinebased	79 (48.2)	73 (40.8)	29 (42.6)	0.38	82 (48.5)	99 (40.9)	0.13
gemcitabinebased	85 (51.8)	106 (59.2)	39 (57.4)		87 (51.5)	143 (59.1)	
UICC stage (2017)							
stage IA	6 (3.7)	12 (6.7)	6 (8.8)	0.37	8 (4.7)	16 (6.6)	0.92
stage IB	33 (20.1)	32 (17.9)	16 (23.5)		33 (19.5)	48 (19.8)	
stage IIA	16 (9.8)	14 (7.8)	5 (7.4)		16 (9.5)	19 (7.9)	
stage IIB	64 (39.0)	54 (30.2)	18 (26.5)		56 (33.1)	80 (33.1)	
stage III	25 (15.2)	41 (22.9)	16 (23.5)		32 (18.9)	50 (20.7)	
stage IV	20 (12.2)	26 (14.5)	7 (10.3)		24 (14.2)	29 (12.0)	
pT (2017)							
pT1a	1 (0.6)	0 (0.0)	2 (2.9)	0.06	0 (0.0)	3 (1.2)	0.36
pT1b	1 (0.6)	4 (2.2)	0 (0.0)		2 (1.2)	3 (1.2)	
pT1c	11 (6.7)	26 (14.5)	8 (11.8)		14 (8.3)	31 (12.8)	
pT2	104 (63.4)	99 (55.3)	40 (58.8)		106 (62.7)	137 (56.6)	
pT3	46 (28.0)	46 (25.7)	18 (26.5)		46 (27.2)	64 (26.4)	
pT4	1 (0.6)	4 (2.2)	0 (0.0)		1 (0.6)	4 (1.7)	
pN (2017)							
pN0	62 (37.8)	71 (39.7)	31 (45.6)	0.18	69 (40.8)	95 (39.3)	0.88
pN1	68 (41.5)	62 (34.6)	17 (25.0)		61 (36.1)	86 (35.5)	
pN2	34 (20.7)	46 (25.7)	20 (29.4)		39 (23.1)	61 (25.2)	
R-status							
0	106 (64.6)	121 (67.7)	48 (70.6)	0.66	108 (63.9)	167 (69.0)	0.28
1	58 (35.4)	58 (32.4)	20 (29.4)		61 (36.1)	75 (31.0)	
grade group							
G1-G2	45 (27.4)	53 (29.6)	23 (33.8)	0.62	51 (30.2)	70 (28.9)	0.78
G3-G4	119 (72.6)	126 (70.4)	45 (66.2)		118 (69.8)	172 (71.1)	

## Data Availability

The expression datasets (both accessed on 11 December 2023) used for validation are publicly accessible on https://portal.gdc.cancer.gov/ and https://dcc.icgc.org/. Raw data on the patient cohorts employed in this study can be obtained from the corresponding author upon reasonable request.

## Supplementary Materials

The following supporting information can be downloaded at: https://www.mdpi.com/article/10.3390/biom15030426/s1, Figure S1: Expression of KRT81, HNF1A and GATA6 in non-neoplastic tissues; Figure S2: Transcriptional subtypes are associated with prognosis in advanced pancreatic cancer patients; Figure S3: Transcriptional subtypes are associated with prognosis in resected pancreatic cancer patients; Figure S4: Transcriptional subtype is associated with therapy response in resected pancreatic cancer patients treated with adjuvant gemcitabine-based chemotherapy; Figure S5: Transcriptional subtypes show differential expression of gemcitabine-resistance promoting genes; Figure S6: KRT81 / HNF1A- or GATA6-based transcriptional subtypes are not associated with specific molecular alterations in pancreatic cancer; Table S1: Prognostic implication of the predominant transcriptional subtype in advanced and resected PDAC patients; Table S2: Comparison of both subtyping panels in the advanced and the resected PDAC cohorts; Table S3: Assessment of transcriptional subtypes in different tissue samples for KRT81 / HNF1A and GATA6; Table S4: Subtype changes during disease progression; Table S5: Comparison of primary tumor subtype with metastatic occurrence and subtype changes for KRT81 / HNF1A and GATA6; Table S6: Transcriptional subtypes in primary tumors and corresponding metastasis; Table S7: Metastatic localization, primary tumor subtype and occurrence of subtype change; Table S8: Multivariate Cox regression analysis of PFS- and OS-associated factors in the advanced pancreatic cancer study cohort; Table S9: Multivariate Cox regression analysis of PFS- and OS-associated factors in the aPDAC cohort stratified for subtypes; Table S10: Multivariate Cox regression analysis of DFS- and OS-associated factors in the resected PDAC cohort; Table S11: Five-year survival rates in the rPDAC cohort for subtypes based on KRT81 / HNF1A- and GATA6- expression according to adjuvant gemcitabine treatment; Table S12: Multivariate Cox regression analysis of DFS- and OS-associated factors in the rPDAC cohort stratified for transcriptional subtypes; Table S13: Study overview on transcriptional subtyping in PDAC.

## Abbreviations

aPDAC	advanced pancreatic ductal adenocarcinoma
aGC	adjuvant gemcitabine
CTX	chemotherapy
DFS	disease-free survival
FFPE	formalin fixed paraffin embedded
FFX	folfirinox
HR	hazard ratio
ICGC	International Cancer Genome Consortium
IHC	immunohistochemistry
naGC	no adjuvant gemcitabine
OS	overall survival
PDAC	pancreatic ductal adenocarcinoma
PFS	progression-free survival
pGC	palliative gemcitabine-based chemotherapy
pnGC	palliative non-gemcitabine-based chemotherapy
rPDAC	resected pancreatic ductal adenocarcinoma
RCT	randomized controlled trial
RNAseq	RNA sequencing
TCGA	the cancer genome atlas
