# Supplementary material for: The Prognostic, Predictive and Clinicopathological Implications of KRT81/HNF1A- and GATA6-Based Transcriptional Subtyping in Pancreatic Cancer
Source: Biomolecules. 2025 Mar 17;15(3):426. doi: 10.3390/biom15030426 (PMC11940166; doi:10.3390/biom15030426)
Supplement: Supplementary file 1 [file biomolecules-15-00426-s001.zip › Table_S1.pdf]

	advanced PDAC cohort										resected PDAC cohort											
subtype	n	%	OS (months)	p-value (log-rank)	HR	95% CI	PFS (months)	p-value (log-rank)	HR	95% CI	n	%	OS (months)	p (log-rank)	HR	95% CI	n	%	DFS (months)	p-value (log-rank)	HR	95% CI
HNF1A pos.	28	20.1	8.4	0.06	1.13	0.97-1.31	3.8	0.01	1.05	0.89-1.23	49	11.9	22.3	0.49	1.07	0.95-1.19	35	11.3	9.5	0.76	1.04	0.92-1.18
HNF1A pos. (double pos.)	11	7.9	17.8				15.2				19	4.6	34.8				17	5.5	13.8			
double neg.	50	36.0	9.1				4.1				179	43.6	19.3				132	42.7	12.4			
KRT81 pos.	38	27.3	6.8				3.6				154	37.5	17.1				116	37.5	9.5			
KRT81 pos. (double pos.)	12	8.6	6.8				2.9				10	2.4	15.5				9	2.9	16.8			
