# Supplementary material for: The Prognostic, Predictive and Clinicopathological Implications of KRT81/HNF1A- and GATA6-Based Transcriptional Subtyping in Pancreatic Cancer
Source: Biomolecules. 2025 Mar 17;15(3):426. doi: 10.3390/biom15030426 (PMC11940166; doi:10.3390/biom15030426)
Supplement: Supplementary file 1 [file biomolecules-15-00426-s001.zip › Table_S10.pdf]

	OS			
	parameter	p-value (Cox)	HR	95%CI
KRT81 / HNF1A	HNF1A-pos.	0.04		
	double-neg.	0.43	1.15	0.82 - 1.60
	KRT81-pos.	0.03	1.46	1.04 - 2.05
	pT1a	0.006		
	pT1b	0.81	0.84	0.19 - 3.67
	pT1c	0.33	0.54	0.16 - 1.85
	pT2	0.44	0.62	0.19 - 2.06
	pT3	0.99	1.00	0.30 - 3.32
	pT4	0.44	0.55	0.12 - 2.51
	R-status	< 0.001	1.51	1.19 - 1.91
	grade group	< 0.001	1.64	1.27 - 2.12
	pN0	< 0.001		
	pN1	0.14	1.22	0.94 - 1.58
	pN2	< 0.001	2.12	1.59 - 2.83
	adjvant gemcitabine treatment	< 0.001	0.47	0.38 - 0.60
GATA6	GATA6-pos.	0.006	0.73	0.58 - 0.91
	pT1a	0.006		
	pT1b	0.88	0.90	0.21 - 3.81
	pT1c	0.33	0.56	0.17 - 1.86
	pT2	0.48	0.66	0.20 - 2.13
	pT3	0.95	1.04	0.32 - 3.40
	pT4	0.52	0.61	0.14 - 2.71
	R-Status	0.001	1.49	1.17 - 1.88
	grade group	<0.001	1.65	1.28 - 2.13
	pN0	<0.001		

	pN1	0.11	1.24	0.95 - 1.60
	pN2	<0.001	2.15	1.62 - 2.87
	adjvant gemcitabine treatment	<0.001	0.47	0.38 - 0.60
	DFS			
	parameter	p-value (Cox)	HR	95%CI
KRT81 / HNF1A	pT1a	0.009		
	pT1b	0.81	1.25	0.21 - 7.51
	pT1c	0.99	0.99	0.23 - 4.26
	pT2	0.68	1.35	0.33 - 5.58
	pT3	0.29	2.18	0.52 - 9.15
	pT4	0.87	1.15	0.20 - 6.61
	grade group	0.02	1.40	1.07 - 1.84
	pN0	< 0.001		
	pN1	0.35	1.15	0.86 - 1.55
	pN2	< 0.001	1.94	1.39 - 2.72
	adjvant gemcitabine treatment	< 0.001	0.59	0.45 - 0.77
GATA6	pT1a	0.009		
	pT1b	0.81	1.25	0.21 - 7.51
	pT1c	0.99	0.99	0.23 - 4.26
	pT2	0.68	1.35	0.33 - 5.58
	pT3	0.29	2.18	0.52 - 9.15
	pT4	0.87	1.15	0.20 - 6.61
	grade group	0.02	1.40	1.07 - 1.84
	pN0	< 0.001		
	pN1	0.35	1.15	0.86 - 1.55

	pN2	< 0.001	1.94	1.39 - 2.72
	adjvant gemcitabine treatment	< 0.001	0.59	0.45 - 0.77
