# Supplementary material for: The Prognostic, Predictive and Clinicopathological Implications of KRT81/HNF1A- and GATA6-Based Transcriptional Subtyping in Pancreatic Cancer
Source: Biomolecules. 2025 Mar 17;15(3):426. doi: 10.3390/biom15030426 (PMC11940166; doi:10.3390/biom15030426)
Supplement: Supplementary file 1 [file biomolecules-15-00426-s001.zip › Table_S11.pdf]

| | | adjuvant gemcitabine treatment | | |
|---|---|---|---|---|
| | 5a survival rate | no | yes | p-value (χ2) |
| GATA6 neg. | dead | 77 (98.7) | 54 (73.0) | <0.001 |
| | alive | 1 (1.3) | 20 (27.0) | |
| GATA6 pos. | dead | 78 (90.7) | 103 (88.0) | 0.55 |
| | alive | 8 (9.3) | 14 (12.0) | |
| KRT81 pos. | dead | 74 (97.4) | 57 (78.1) | <0.001 |
| | alive | 2 (2.6) | 16 (21.9) | |
| double neg. | dead | 56 (91.8) | 75 (84.3) | 0.17 |
| | alive | 5 (8.2) | 14 (15.7) | |
| HNF1A pos. | dead | 25 (92.6) | 25 (86.2) | 0.44 |
| | alive | 2 (7.4) | 4 (13.8) | |
