# Supplementary material for: The Prognostic, Predictive and Clinicopathological Implications of KRT81/HNF1A- and GATA6-Based Transcriptional Subtyping in Pancreatic Cancer
Source: Biomolecules. 2025 Mar 17;15(3):426. doi: 10.3390/biom15030426 (PMC11940166; doi:10.3390/biom15030426)
Supplement: Supplementary file 1 [file biomolecules-15-00426-s001.zip › Table_S12.pdf]

	DFS			
	parameter	p-value (Cox)	HR	95%CI
GATA6 neg.	R-status	0.08	1.51	0.95 - 2.40
	pN0	0.007		
	pN1	0.03	0.58	0.36 - 0.96
	pN2	0.12	1.53	0.89 - 2.62
	adjuvant gemcitabine treatment	<0.001	0.28	0.18 - 0.44
GATA6 postive	pT1a	0.01		
	pT1b	0.47	0.55	0.11 - 2.75
	pT1c	0.14	0.39	0.11 - 1.34
	pT2	0.12	0.39	0.12 - 1.28
	pT3	0.56	0.70	0.21 - 2.34
	pT4	0.45	0.54	0.11 - 2.63
	grade group	<0.001	1.99	1.41 - 2.82
	pN0	<0.001		
	pN1	0.02	1.53	1.09 - 2.16
	pN2	<0.001	2.22	1.53 - 3.24
KRT81 pos.	UICC stage IA	0.006		
	UICC stage IB	0.66	1.25	0.46 - 3.38
	UICC stage IIA	0.16	2.26	0.72 - 7.14
	UICC stage IIB	0.178	1.93	0.74 - 5.03
	UICC stage III	0.02	3.30	1.18 - 9.23
	UICC stage IV	0.007	4.67	1.51 - 14.43
	R-status	0.003	2.02	1.28 - 3.20
	adjuvant gemcitabine treatment	<0.001	0.29	0.19 - 0.45
double neg.	pT1c	0.03		
	pT1c	0.95	0.95	0.20 - 4.43
	pT2	0.91	0.91	0.18 - 4.51
	pT3	0.30	2.36	0.46 - 12.04
	pT4	0.34	2.49	0.38 - 16.53
	UICC stage IA	0.009		
	UICC stage IB	0.53	1.45	0.46 - 4.57
	UICC stage IIA	0.43	0.60	0.17 - 2.15
	UICC stage IIB	0.78	0.86	0.31 - 2.43
	UICC stage III	0.18	2.14	0.71 - 6.43
	UICC stage IV	0.48	0.67	0.22 - 2.05
	grade group	0.09	1.46	0.94 - 2.26
HNF1A pos.	pT1a	0.03		
	pT1c	0.86	1.22	0.14 - 10.67
	pT2	0.63	1.65	0.22 - 12.60
	pT3	0.14	4.89	0.61 - 39.53
	pN0	0.02		

	pN1	0.02	2.42	1.14 - 5.13
	pN2	0.02	2.67	1.19 - 5.97
	OS			
	parameter	p-value (Cox)	HR	95%CI
GATA6 neg.	R-status	0.003	1.78	1.22 - 2.59
	pN0	0.02		
	pN1	0.46	0.86	0.58 - 1.28
	pN2	0.03	1.63	1.05 - 2.55
	adjuvant gemcitabine treatment	<0.001	0.24	0.16 - 0.35
GATA6 pos.	pT1a	0,02		
	pT1b	0,66	0,67	0.11 - 4.05
	pT1c	0,08	0,31	0.08 - 1.13
	pT2	0,05	0,29	0.09 - 1.01
	pT3	0,50	0,65	0.18 - 2.30
	pT4	0,58	0,63	0.12 - 3.25
	grade group	0,001	2,19	1.38 - 3.46
	pN0	0,02		
	pN1	0,30	1,29	0.80 - 2.07
	pN2	0,006	2,03	1.23 - 3.36
KRT81 pos.	UICC stage IA	0.004		
	UICC stage IB	0.75	0.85	0.32 - 2.28
	UICC stage IIA	0.28	1.79	0.63 - 5.11
	UICC stage IIB	0.63	1.26	0.50 - 3.19
	UICC stage III	0.20	1.92	0.71 - 5.19
	UICC stage IV	0.05	2.91	1.03 - 8.23
	R-status	<0.001	2.43	1.67 - 3.55
	grade group	0.02	1.59	1.06 - 2.37
	adjuvant gemcitabine treatment	<0.001	0.24	0.17 - 0.35
double neg.	UICC stage IA	0.01		
	UICC stage IB	0.64	1.19	0.58 - 2.46
	UICC stage IIA	0.67	1.20	0.53 - 2.70
	UICC stage IIB	0.93	1.02	0.53 - 2.03
	UICC stage III	0.01	2.38	1.19 - 4.74
	UICC stage IV	0.85	1.08	0.50 - 2.31
	R-status	0.04	1.45	1.01 - 2.07
	grade group	<0.001	1.97	1.33 - 2.91
HNF1A postive	pT1a	0.002		
	pT1c	0.60	0.64	0.13 - 3.34
	pT2	0.59	0.66	0.15 - 2.97
	pT3	0.25	2.49	0.52 - 11.88
	pN0	0.002		
	pN1	0.01	2.57	1.24 - 5.34
	pN2	<0.001	3.27	1.63 - 6.56
