# Supplementary material for: The Prognostic, Predictive and Clinicopathological Implications of KRT81/HNF1A- and GATA6-Based Transcriptional Subtyping in Pancreatic Cancer
Source: Biomolecules. 2025 Mar 17;15(3):426. doi: 10.3390/biom15030426 (PMC11940166; doi:10.3390/biom15030426)
Supplement: Supplementary file 1 [file biomolecules-15-00426-s001.zip › Table_S13.pdf]

marker	author	year	cohort	clinical situation	n=	TNM version	detection method	antibodies, dilution	tissue type	scoring method	cutoff definition	results	cohort stratification based on therapy	clinical relevance/conclusion
GATA6	Martinelli	2017	1	resected	313	7th	IHC	polyclonal goat anti-GATA6 antibody (catalog no. AF1700, R&D Systems)<br>dilution 1:1000	TMA	high, medium, low	NR	GATA6 high: 155 (49%)<br>GATA6 medium: 121 (39%)<br>GATA6 low: 37 (12%)	5FU / leucovorin vs. gemcitabine	borderline prognostic with 5FU / leucovorin treatment, not with gemcitabine<br>correlates with tumor diffentiation
	O'Kane	2021	1	advanced stage	106	NA	ISH, IHC	polyclonal goat anti-GATA6 antibody (catalog no. AF1700, R&D Systems)<br>dilution 1:1000	resections, biopsies	semiquantitative score (1-4)	score 1: weak nuclear staining in at least 5% tumor cells<br>score 2: moderate nuclear staining in the tumor<br>score 3: strong nuclear immunopositivity<br>score 4. very strong and diffuse staining	GATA6 high: 78 (74%)	NR	prognostic<br>IHC correlates with ISH
	Duan	2021	1	advanced stage	130<br>(106 patients already reported by O´Kane et al.)	NA	IHC	polyclonal goat anti-GATA6 antibody (catalog no. AF1700, R&D Systems)<br>dilution 1:1000	biopsies, whole mount	low / high	low: semiquantitative score 1-2<br>high: semiqunaititative score 3-4	GATA6 high: 73 (56%)	palliative modified FOLFIRINOX vs. gemcitabine–nab-paclitaxel. 10 patients did not receive any treatment	predictive for palliative mFOLFIRINOX but not for gemcitabine/nab-paclitaxel<br>digital assessment may improve scoring
	de Andrés	2021	1	resected	745<br>(282 patients from the ESPAC-3 trial already reported by Martinelli et al. 2017)	7th	IHC	polyclonal goat anti-GATA6 antibody (R&D systems, AF1700, 0.2ug/mL)<br>dilution NR	TMA	low / high	histoscore quantification (proportion of positive tumor cells multiplied by intensitiy from 0 to 3)<br>low: Histoscore < 150<br>high: Histoscore ≥ 150	GATA6 high: 380 (51%)	NR	prognostic
	Beutel	2021	1	advanced stage	25 PDO and 21 corresponding tissue from liver metastases	NA	IHC	monoclonal rabbit anti-GATA6 antibody (clone D61E4, Cell Signaling Technology)<br>dilution 1:100	PDO´s, biopsies	NR	NR	GATA6 high tissue: 18 (85.7%)<br>GATA6 high PDO: 19 (76.0%)	NR	not prognostic (PFS)<br>differential expression between tissue and PDO in 4/21 (19%)
	Shoucair	2022	1	resected	76	NR	IHC	NR	NR	low/high	NR	GATA6 high: 36 (47.3%)	adjuvant treatment vs. no adjuvant treatment	predictive for adjuvant therapy
	Heredia-Soto	2023	1	resected	89	7th	IHC	polyclonal goat anti-GATA6 antibody (catalog no. AF1700, R&D Systems)<br>dilution NR	TMA	low/high	histoscore quantification (proportion of positive tumor cells multiplied by intensitiy from 0 to 3)<br>low: Histoscore < 30<br>high: Histoscore ≥ 30	GATA6 high: 72 (81%)	only adjuvant gemcitabine treated patients included	prognostic (DFS)<br>correlates with CA19-9 levels<br>PanIN and tumor tissue comparable
	Guenther	2024	1	resected	411	8th	IHC	polyclonal rabbit anti-GATA6 antibody (PA1-104, Thermo Fisher, Germering, Germany)<br>dilution 1:200	TMA	negative / positive	tumors with distinct nuclear staining were considered GATA6 positive	GATA6 positive 242 (58.9%)	gemcitabine-based vs. non gemcitabine-based	no relevant associations to clinicopathological variables<br>prognostic in resected PDAC<br>predictive for gemcitabine-based adjvant treatment
														influences the role of clinicophatological variables such as R-status

			2	advanced stage	139	NA			TMA			GATA6 positive<br>55 (39.9%)	gemcitabine-based vs. non gemcitabine-based	not prognostic. Predictive for gemcitabine-based palliative treatment<br>no associations to clinicopathological variables
			3	resected	57	8th			TMA			GATA6 positive<br>33 (57.9%)	NA	subtype may switch during metastatic progression<br>subtype switch more frequent in synchronous metastasis<br>strong correlation between primary and metastasis subtype<br>tendency to switch to a prognostically favorable subtype
KRT81	Park	2022	1	resected	108	8th	IHC	mouse monoclonal anti-keratin 81 antibody (catalog no. SC-100929, Santa Cruz Biotechnology Inc.)<br>dilution 1:500	TMA	negative / positive	"KRT81-positive": >30% KRT81 expressing tumor cells	KRT81+: 30 (28%)<br>DP: NR	NR	prognostic<br>corrrelates with reduced gland formation
			2	resected<br>neoadjuvant treatment	68	8th	IHC		TMA			KRT81+: 30 (25%)<br>DP: NR	NR	associated with remaing residual tumor
			3	resected n=12<br>advanced disease n= 43	55	8th	IHC		NR			KRT81+:24(44%)<br>NA: 18 (33%)	NR	prognostic
HNF1a	Lu	2019	1	resected	128	8th	IHC	rabbit anti-HNF1A antibody (catalog no. ab96777, Abcam)<br>dilution 1:200	NR	semiquantitative score according to IRS	low: 0-2<br>moderate: 3-6<br>high: 8-12	HNF1A low:70 (55%)<br>HNF1A moderate: 37 (29%)<br>HNF1A high: 21 (16%)	NA	prognostic in gemcitabine treated patients<br>correlates with stage and Pn1
	Park	2022	1	resected	108	8th	IHC	anti–HNF1A antibody (catalog no. ab96777, Abcam),<br>dilution1:500	TMA	negative / positive	"HNF1A-positive": nuclear expression, any intensity, moderate or strong intensity, ≥ 5% of cells	HNF1A+: 35 (32%)<br>DP:NR	NR	not prognostic<br>corrrelates with increased gland formation
			2	resected<br>neoadjuvant treatment	68	8th			TMA			NR	NR	not associated with remaining residual tumor
			3	resected n=12<br>advanced disease n= 43	55	8th			TMA			NR	NR	NR
HNF1a + KRT81	Noll	2016	1	resected	231	7th	IHC	mouse monoclonal anti-keratin 81 antibody (catalog no. sc-100929, Santa Cruz Biotechnology Inc.)<br>dilution NR<br>rabbit polyclonal anti–HNF1A antibody (catalog no. sc-8986, Santa Cruz Biotechnology Inc)<br>dilution NR	TMA	negative / positive	"KRT81-positive": detectable staining regardless of signal strength / number of positive cells,<br>strong expression, >10% KRT81 expressing tumor cells<br>"HNF1A-positive": detectable staining regardless of signal strength / number of positive cells,<br>double positive cases excluded	DN: 92 (40%),<br>KRT81+: 79 (34%)<br>HNF1A+: 46 (20%)<br>DP: 14 (6%)	NR	prognostic (HNF1A / KRT81). correlates with grading
	Muckenhuber	2018	1	resected	262	7th	IHC	mouse monoclonal anti-keratin 81 antibody (catalog no. sc-100929, Santa Cruz Biotechnology Inc.)<br>dilution 1:200<br>rabbit polyclonal anti–HNF1A antibody (catalog no. sc-8986, Santa Cruz Biotechnology Inc)<br>dilution 1:200	TMA	negative / positive	"KRT81-positive": >30% KRT81 expressing tumor cells<br>"HNF1A-positive": medium to strong nuclear staining of HNF1A<br>double positive cases excluded	DN: 165 (63%),<br>KRT81+: 59 (23%)<br>HNF1A+: 35 (13%)<br>DP: 3 (1%)	adjuvant treatment vs. no adjuvant treatment	prognostic (HNF1A / KRT81)<br>correlates with sex

			1	resected	130	7th		mouse monoclonal anti-keratin 81 antibody (catalog no. sc-100929, Santa Cruz Biotechnology Inc.)<br>dilution 1:500<br>rabbit polyclonal anti–HNF1A antibody (catalog no. sc-8986, Santa Cruz Biotechnology Inc)<br>dilution 1:100	TMA	NR	NR	DN: 41 (32%),<br>KRT81+: 21 (16%)<br>HNF1A+: 50 (39%)<br>DP: 18 (13%)	adjuvant treatment vs. no adjuvant treatment	prognostic (HNF1A / KRT81)<br>no correlation to clinicopathological variables
			1	advanced stage	125	NA			TMA	NR	NR	DN: 62 (50%),<br>KRT81+: 11 (9%)<br>HNF1A+: 47 (38%)<br>DP: 5 (3%)	gemcitabine-based vs. FOLFIRINOX-based	prognostic (HNF1A / KRT81)<br>no correlation to clinicopathological variables<br>HNF1A+ increased initial tumor control in folfirinox-based treatment
	Kaissis	2019	1	resected	55<br>(21 Patients derived from Muckenhuber et al.)	7th	IHC	according to Muckenhuber et al.	NR/TMA	according Muckenhuber et al.	according to Muckenhuber et al.<br>double negative and double positive cases were excluded	KRT81+: 27 (49%)<br>DN: NR<br>HNF1A+: 28 (51%)<br>DP: NR	FOLFIRINOX-based palliative treatment vs. gemcitabine-based palliative treatment	prognostic<br>no correlation with clinicopathological variables<br>KRT81- predictive for palliative FOLFIRINOX.<br>KRT81+ predictive for palliative gemcitabine
	Kaissis	2020	1	resected	103<br>(patients derived from Muckenhuber et al.)	7th	IHC	according to Muckenhuber et al.	NR/TMA	according Muckenhuber et al.	according to Muckenhuber et al.	KRT81+: 16 (15.5%)<br>DN: NR<br>HNF1A+: NR<br>DP: NR	NR	prognostic
	Kruger	2022	1	resected	71	7th	IHC	anti-KRT81, (clone 3B10-5B10, LS Bio, Seattle, WA)<br>dilution 1:120<br>polyclonal rabbit anti-HNF1A (Atlas antibodies, Stockholm, Sweden)<br>dilution 1:100	resections	negative / positive	"HNF1A-positive": strong and specific nuclear staining<br>"KRT81-positive": strong and specific membranous and cytoplasmatic staning	M1-PUL: KRT81+: 11 (29%)<br>DN: 19 (50%)<br>HNF1A+: 8 (21%)<br>DP: 0 (0%)<br>M1-ANY:<br>KRT81+: 8 (24%)<br>DN: 20 (61%)<br>HNF1A+: 5 (15%)<br>DP: 0 (0%)	NR	no significant differences between isolated lung and other metastatic patterns
	Peng	2023	1	resected	57	not stated	IHC	mouse monoclonal anti-keratin 81 antibody (catalog no. sc-100929, Santa Cruz Biotechnology Inc.)<br>dilution 1:200<br>mouse monoclonal anti–HNF1A antibody (catalog no.sc-393925, Santa Cruz Biotechnology Inc)<br>dilution 1:200	resections	negative / positive	NR	HNF1A+: 31 (54%)<br>KRT81+: 19 (33%)<br>DN: 7 (12%)	NR	NR

	Guenther	2024	1	resected	411	8th	IHC	anti-KRT81, (clone 3B10-5B10, LS Bio, Seattle, WA)<br>dilution 1:120<br>polyclonal rabbit anti-HNF1A (Atlas antibodies, Stockholm, Sweden)<br>dilution 1:100	TMA	negative / positive	samples with ≥ 30% KRT81- or HNF1A-positive tumor cells: positive for each marker<br>double positive samples categorized according to predominant marker	KRT81+: 154 (37.5)<br>HNF1A+: 49 (11.9)<br>DP: 29 (7.0)<br>DN: 179 (43.6)	gemcitabine-based vs. non gemcitabine-based	prognostic in rPDAC (KRT81 / HNF1A)<br>no associations to clinicopathological variables<br>predictive for adjuvant gemcitabine<br>influences clinicopathological risk factors such as R-status
			2	advanced stage	139	NA			TMA			KRT81+: 38 (27.3%)<br>HNF1A+: 28 (20.1%)<br>DP: 23 (16.6%)<br>DN: 50 (36.0)	gemcitabine-based vs. non gemcitabine-based	not prognostic in aPDAC<br>predictive for gemcitabine<br>no relevant associations to clinicopathological variables
			3	resected	57	8th			TMA			KRT81+: 39 (68.4%)<br>HNF1A+: 0 (0.0%)<br>DP: 5 (8.8%)<br>DN: 13 (22.8.0)	NA	subtype may switch during metastatic progression<br>subtype switch independent of synchronous / metachronous metastasis<br>strong correlation between primary and metastasis subtype<br>tendency to switch to a prognostically favorable subtype
