# Supplementary material for: The Prognostic, Predictive and Clinicopathological Implications of KRT81/HNF1A- and GATA6-Based Transcriptional Subtyping in Pancreatic Cancer
Source: Biomolecules. 2025 Mar 17;15(3):426. doi: 10.3390/biom15030426 (PMC11940166; doi:10.3390/biom15030426)
Supplement: Supplementary file 1 [file biomolecules-15-00426-s001.zip › Table_S2.pdf]

	aPDAC cohort			rPDAC cohort		
	GATA6 neg.	GATA6 pos.	p-value (χ2-test)	GATA6 neg.	GATA6 pos.	p-value (χ2-test)
HNF1A pos.	14 (16.7)	25 (45.5)	<0.001	7 (4.1)	61 (25.2)	< 0.001
double neg.	37 (44.0)	13 (23.6)		66 (39.1)	113 (46.7)	
KRT81 pos.	33 (39.3)	17 (30.9)		96 (56.8)	68 (28.1)	
