# Supplementary material for: The Prognostic, Predictive and Clinicopathological Implications of KRT81/HNF1A- and GATA6-Based Transcriptional Subtyping in Pancreatic Cancer
Source: Biomolecules. 2025 Mar 17;15(3):426. doi: 10.3390/biom15030426 (PMC11940166; doi:10.3390/biom15030426)
Supplement: Supplementary file 1 [file biomolecules-15-00426-s001.zip › Table_S3.pdf]

		subtype repunched			
		HNF1a pos.	double neg.	KRT81 pos.	p-value (χ2-test)
subtype 1st assessment	HNF1a pos.	2 (100.0)	0 (0.0)	1 (7.7)	<0.001
	double neg.	0 (0.0)	8 (80.0)	4 (30.8)	
	KRT81 pos.	0 (0.0)	2 (20.0)	8 (61.5)	
		GATA6 neg.	GATA6 pos.	p-value (χ2-test)	
	GATA6 neg.	7 (87.%)	1 (5.9)	<0.001	
	GATA6 pos.	1 (12.5)	16 (94.1)		
