# Supplementary material for: The Prognostic, Predictive and Clinicopathological Implications of KRT81/HNF1A- and GATA6-Based Transcriptional Subtyping in Pancreatic Cancer
Source: Biomolecules. 2025 Mar 17;15(3):426. doi: 10.3390/biom15030426 (PMC11940166; doi:10.3390/biom15030426)
Supplement: Supplementary file 1 [file biomolecules-15-00426-s001.zip › Table_S4.pdf]

	KRT81/HNF1A			GATA6		
	subtype switch			subtype switch		
metastasis type	no	yes	p-value (χ2-test)	no	yes	p-value (χ2-test)
synchronous	25 (64.1)	8 (44.4)	0.16	20 (48.8)	13 (81.3)	0.03
metachronous	14 (35.9)	10 (55.6)		21 (51.2)	2 (18.8)	
