# Supplementary material for: The Prognostic, Predictive and Clinicopathological Implications of KRT81/HNF1A- and GATA6-Based Transcriptional Subtyping in Pancreatic Cancer
Source: Biomolecules. 2025 Mar 17;15(3):426. doi: 10.3390/biom15030426 (PMC11940166; doi:10.3390/biom15030426)
Supplement: Supplementary file 1 [file biomolecules-15-00426-s001.zip › Table_S5.pdf]

	subtype primary tumor, no (%)				subtype primary tumor, no (%)		
	KRT81 pos. (n=37)	double neg. (n=17)	HNF1A pos. (n=3)	p-value (χ2-test)	GATA6 neg. (n=23)	GATA6 pos. (n=34)	p-value (χ2-test)
subtype change							
no switch	25 (67.6)	13 (76.5)	1 (33.3)	0.33	16 (69.6)	25 (73.5)	0.74
switch	12 (32.4)	4 (23.5)	2 (66.7)		7 (30.4)	9 (26.5)	
metastasis type							
synchronous	23 (62.2)	9 (52.9)	1 (33.3)	0.55	13 (56.5)	20 (58.8)	0.86
metachronous	14 (37.8)	8 (47.1)	2 (66.7)		10 (43.5)	14 (41.2)	
prognostic properties of subtype switch							
subtype constant	25 (68.4)	13 (76.5)	1 (33.3)	<0.001	16 (69.6)	25 (73.5)	<0.001
prognostically more favorable	12 (32.4)	3 (17.6)	0 (0.0)		7 (30.4)	0 (0.0)	
prognostically less favorable	0 (0.0)	1 (5.9)	2 (66.7)		0 (0.0)	9 (26.5)	
