# Supplementary material for: The Prognostic, Predictive and Clinicopathological Implications of KRT81/HNF1A- and GATA6-Based Transcriptional Subtyping in Pancreatic Cancer
Source: Biomolecules. 2025 Mar 17;15(3):426. doi: 10.3390/biom15030426 (PMC11940166; doi:10.3390/biom15030426)
Supplement: Supplementary file 1 [file biomolecules-15-00426-s001.zip › Table_S6.pdf]

		subtype metastasis			
		HNF1a pos.	double neg.	KRT81 pos.	p-value (χ2-test)
subtype primary tumor	HNF1a pos.	1 (16.7)	0 (0.0)	2 (7.1)	<0.001
	double neg.	3 (50.0)	13 (56.5)	1 (3.6)	
	KRT81 pos.	2 (33.3)	10 (43.5)	25 (89.3)	
		GATA6 pos.	GATA6 neg.	p-value (χ2-test)	
	GATA6 pos.	16 (64.0)	7 (21.9)	0.001	
	GATA6 neg.	9 (36.0)	25 (78.1)		
