# Supplementary material for: The Prognostic, Predictive and Clinicopathological Implications of KRT81/HNF1A- and GATA6-Based Transcriptional Subtyping in Pancreatic Cancer
Source: Biomolecules. 2025 Mar 17;15(3):426. doi: 10.3390/biom15030426 (PMC11940166; doi:10.3390/biom15030426)
Supplement: Supplementary file 1 [file biomolecules-15-00426-s001.zip › Table_S7.pdf]

	metastasis localization					
	HEP	PER	PUL	PLE	OTH	p-value (χ2-test)
subtype primary tumor						
HNF1A positive	0 (0.0)	2 (12.5)	0 (0.0)	1 (50.0)	0 (0.0)	0.10
double negative	8 (26.7)	5 (31.3)	2 (50.0)	0 (0.0)	2 (40.0)	
KRT81 positive	22 (73.3)	9 (56.3)	2 (50.0)	1 (50.0)	3 (80.0)	
subtype metastasis						
HNF1A positive	2 (6.7)	3 (18.8)	1 (25.0)	0 (0.0)	0 (0.0)	0.28
double negative	13 (43.3)	4 (25.0)	3 (75.0)	0 (0.0)	3 (60.0)	
KRT81 positive	15 (50.0)	9 (56.3)	0 (0.0)	2 (100.0)	2 (40.0)	
subtype switch (KRT81 / HNF1A)						
no switch	21 (70.0)	11 (68.8)	2 (50.0)	1 (50.0)	4 (80.0)	0.86
switch	9 (30.0)	5 (31.1)	2 (50.0)	1 (50.0)	1 (20.0)	
subtype primary tumor						
GATA6 negative	10 (33.3)	10 (62.5)	2 (50.0)	0 (0.0)	1 (20.0)	0.18
GATA6 positive	20 (66.7)	6 (37.5)	2 (50.0)	2 (100.0)	4 (80.0)	
subtype metastasis						
GATA6 negative	16 (53.3)	7 (43.8)	1 (25.0)	0 (0.0)	1 (20.0)	0.36
GATA6 positive	14 (46.7)	9 (56.3)	3 (75.0)	2 (100.0)	4 (80.0)	
subtype switch (GATA6)						
no switch	22 (73.3)	11 (68.8)	3 (75.0)	2 (100.0)	3 (60.0)	0.87
switch	8 (26.7)	5 (31.3)	1 (25.0)	0 (0.0)	2 (40.0)	
