# Supplementary material for: The Prognostic, Predictive and Clinicopathological Implications of KRT81/HNF1A- and GATA6-Based Transcriptional Subtyping in Pancreatic Cancer
Source: Biomolecules. 2025 Mar 17;15(3):426. doi: 10.3390/biom15030426 (PMC11940166; doi:10.3390/biom15030426)
Supplement: Supplementary file 1 [file biomolecules-15-00426-s001.zip › Table_S8.pdf]

	OS			
	parameter	p-value (Cox)	HR	95%CI
KRT81 / HNF1A	HNF1A pos.	0.07		
	double neg.	1.00	1.00	0.63 - 1.55
	KRT81 pos.	0.06	1.55	1.01 - 2.50
	grade group	0.009	1.64	1.13 - 2.39
	CTX type	0.09	1.85	1.12 - 3.04
	disease stage at therapy initiation	0.02	1.85	1.12 - 3.04
GATA6	grade group	0.002	1.77	1.23 - 2.54
	disease stage at therapy initiation	0.02	1.81	1.10 - 2.98
	PFS			
	parameter	p-value (Cox)	HR	95%CI
KRT81 / HNF1A	grade group	0.002	1.89	1.27 - 2.80
	CTX type	< 0.001	0.43	0.28 - 0.65
	disease stage at therapy initiation	0.04	1.74	1.03 - 2.94
GATA6	grade group	0.002	1.89	1.27 - 2.80
	CTX type	< 0.001	0.43	0.28 - 0.65
	disease stage at therapy initiation	0.04	1.74	1.03 - 2.94
