# Supplementary material for: The Prognostic, Predictive and Clinicopathological Implications of KRT81/HNF1A- and GATA6-Based Transcriptional Subtyping in Pancreatic Cancer
Source: Biomolecules. 2025 Mar 17;15(3):426. doi: 10.3390/biom15030426 (PMC11940166; doi:10.3390/biom15030426)
Supplement: Supplementary file 1 [file biomolecules-15-00426-s001.zip › Table_S9.pdf]

	PFS			
	parameter	p-value (Cox)	HR	95%CI
GATA6 neg.	grade group	0.04	1.79	1.02 - 3.14
	CTX type	< 0.001	0.33	0.18 - 0.59
GATA6 pos.	disease stage at therapy initiation	0.04	2.57	1.04 - 6.34
	grade group	0.06	1.77	0.97 - 3.21
KRT81 pos.	KPS group	0.03	0.45	0.22 - 0.93
	CTX type	0.02	0.41	0.19 - 0.87
	disease stage at therapy initiation	0.06	2.31	0.96 - 5.60
double neg.	CTX type	< 0.001	0.23	0.11 - 0.47
	disease stage at therapy initiation	0.07	2.47	0.94 - 6.49
HNF1A pos.	grade group	0.013	2.59	1.22 - 5.49
	OS			
	parameter	p-value (Cox)	HR	95%CI
GATA6 neg.	grade group	0.002	2.17	1.33 - 3.54
GATA6 pos.	disease stage at therapy initiation	0.10	2.00	0.89 - 4.54
KRT81 pos.	KPS group	0.007	0.42	0.22 - 0.79
	disease stage at therapy initiation	0.04	2.40	1.05 - 5.51
double neg.	CTX type	0.04	0.52	0.28 - 0.96
HNF1A pos.	grade group	0.07	1.88	0.95 - 3.71
