# Supplementary figures and images for: The Prognostic, Predictive and Clinicopathological Implications of KRT81/HNF1A- and GATA6-Based Transcriptional Subtyping in Pancreatic Cancer
Source: Biomolecules. 2025 Mar 17;15(3):426. doi: 10.3390/biom15030426 (PMC11940166; doi:10.3390/biom15030426)

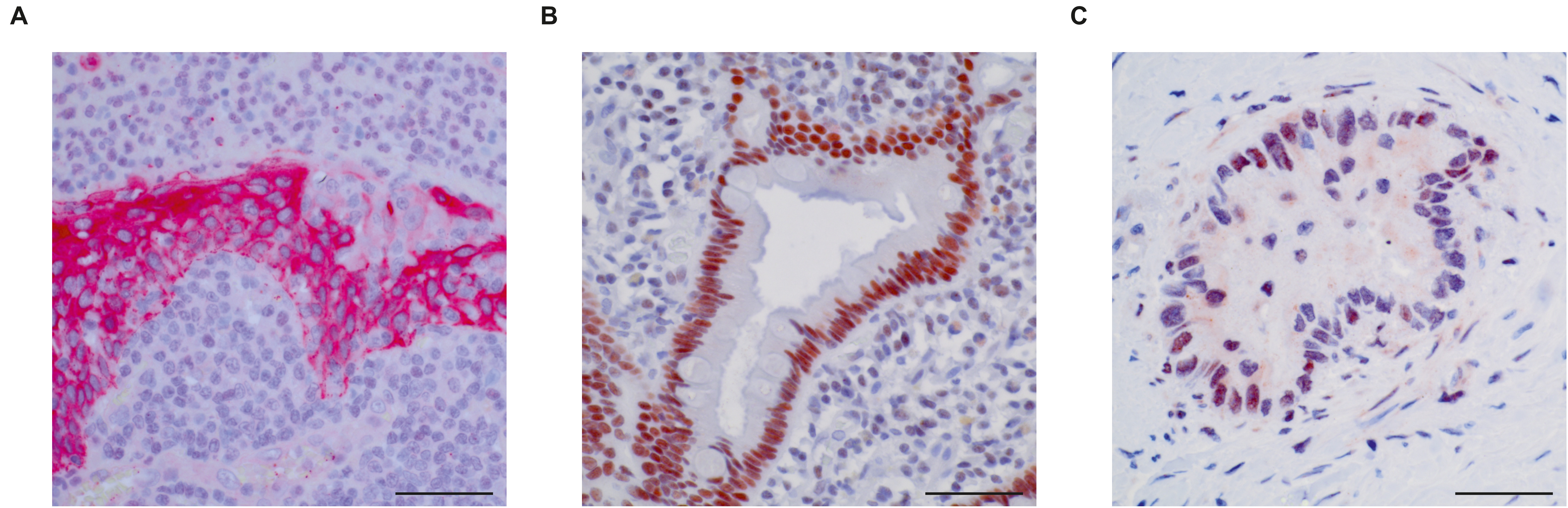

Supplement: Supplementary file 1 [file biomolecules-15-00426-s001.zip › figure_S1.jpg]

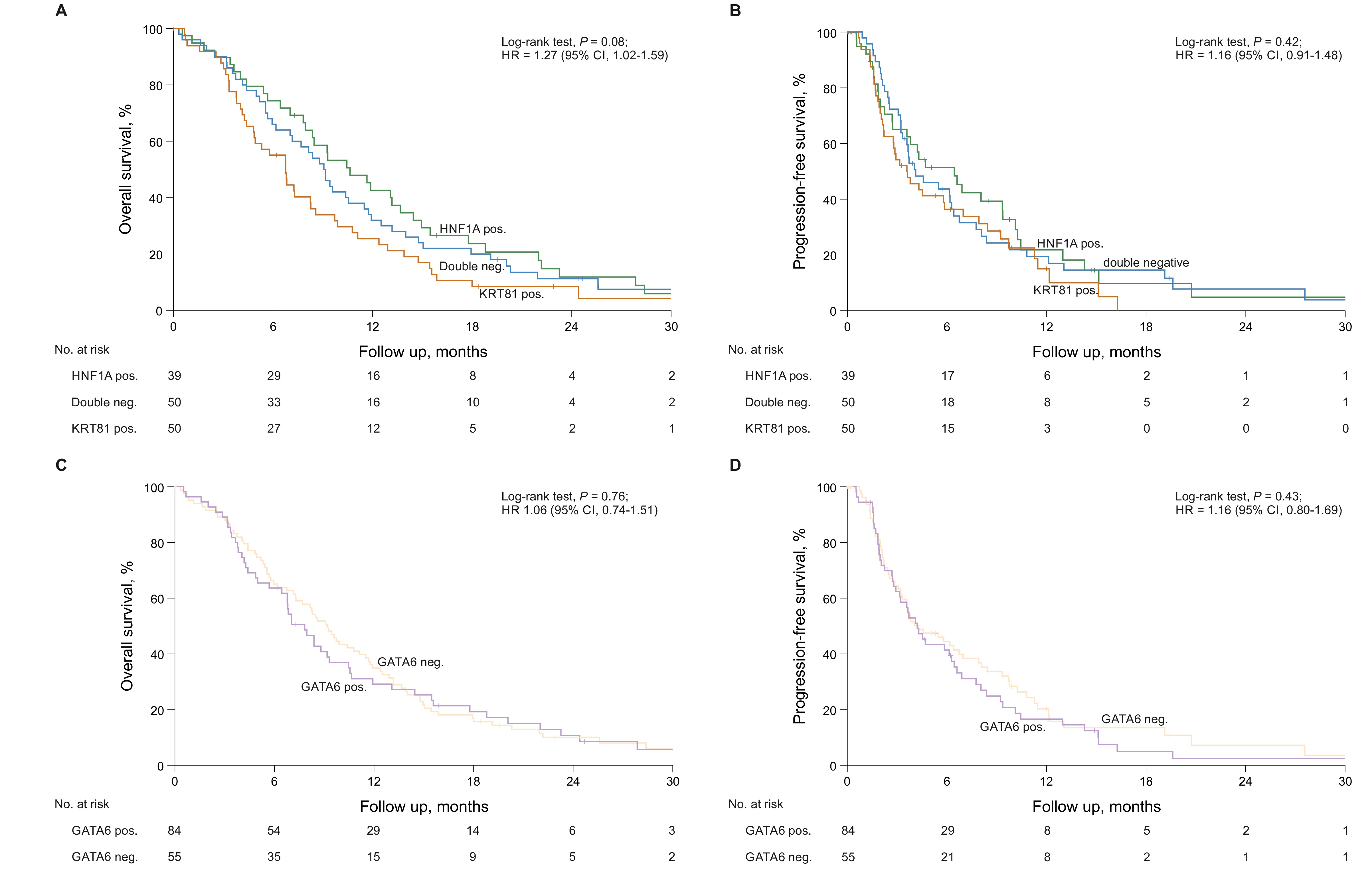

Supplement: Supplementary file 1 [file biomolecules-15-00426-s001.zip › figure_S2.jpg]

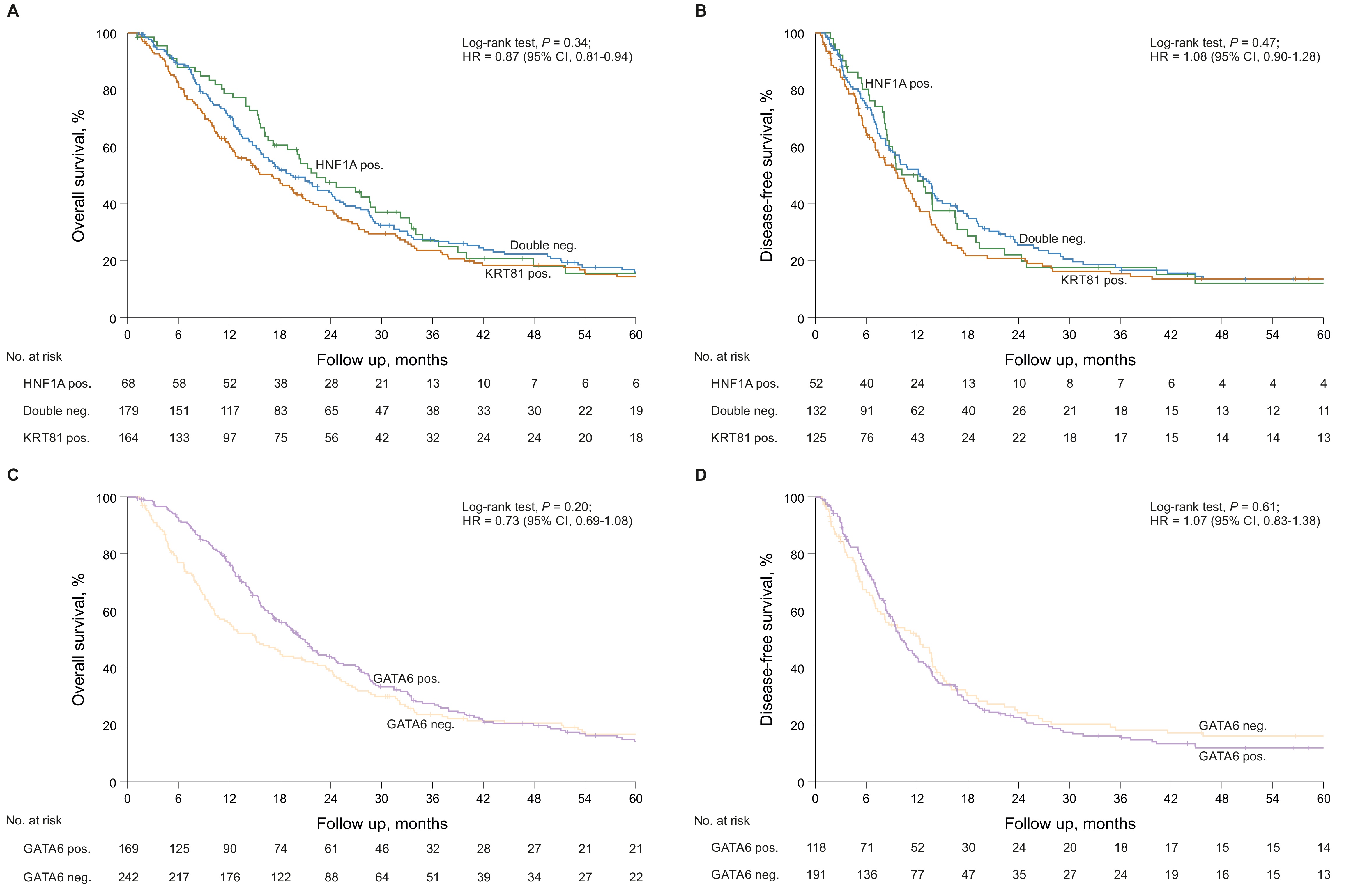

Supplement: Supplementary file 1 [file biomolecules-15-00426-s001.zip › figure_S3.jpg]

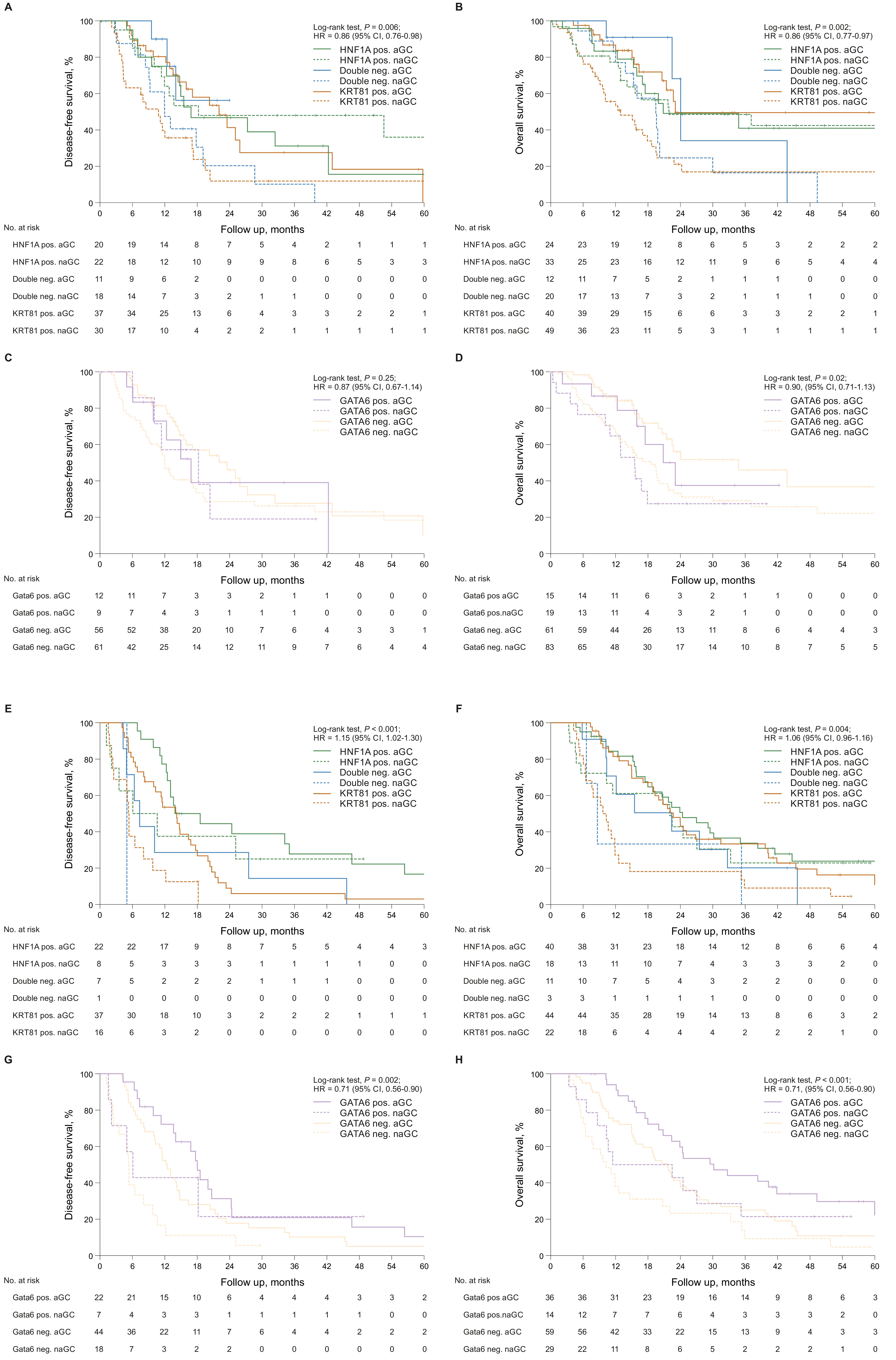

Supplement: Supplementary file 1 [file biomolecules-15-00426-s001.zip › figure_S4.jpg]

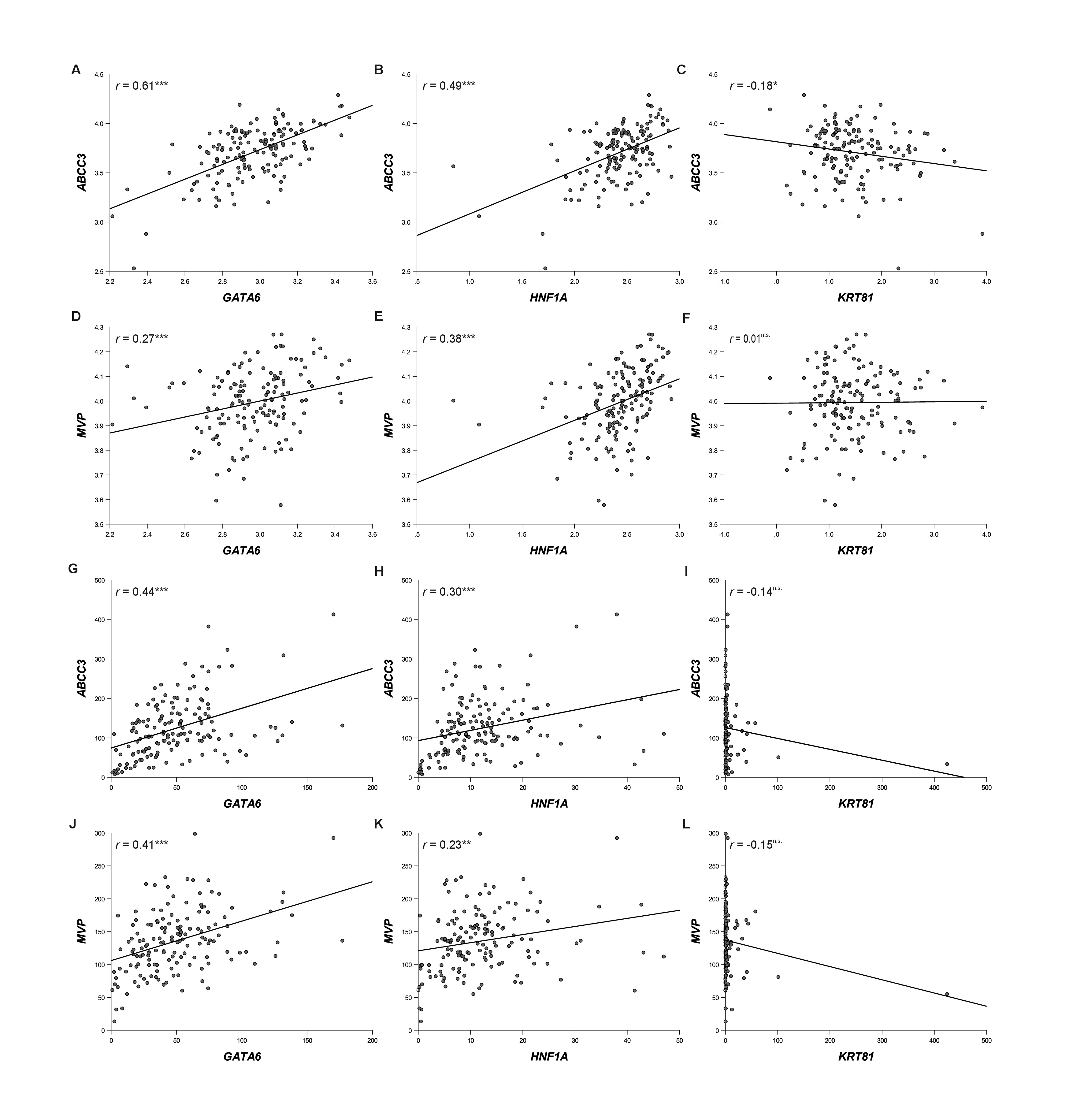

Supplement: Supplementary file 1 [file biomolecules-15-00426-s001.zip › figure_S5.jpg]

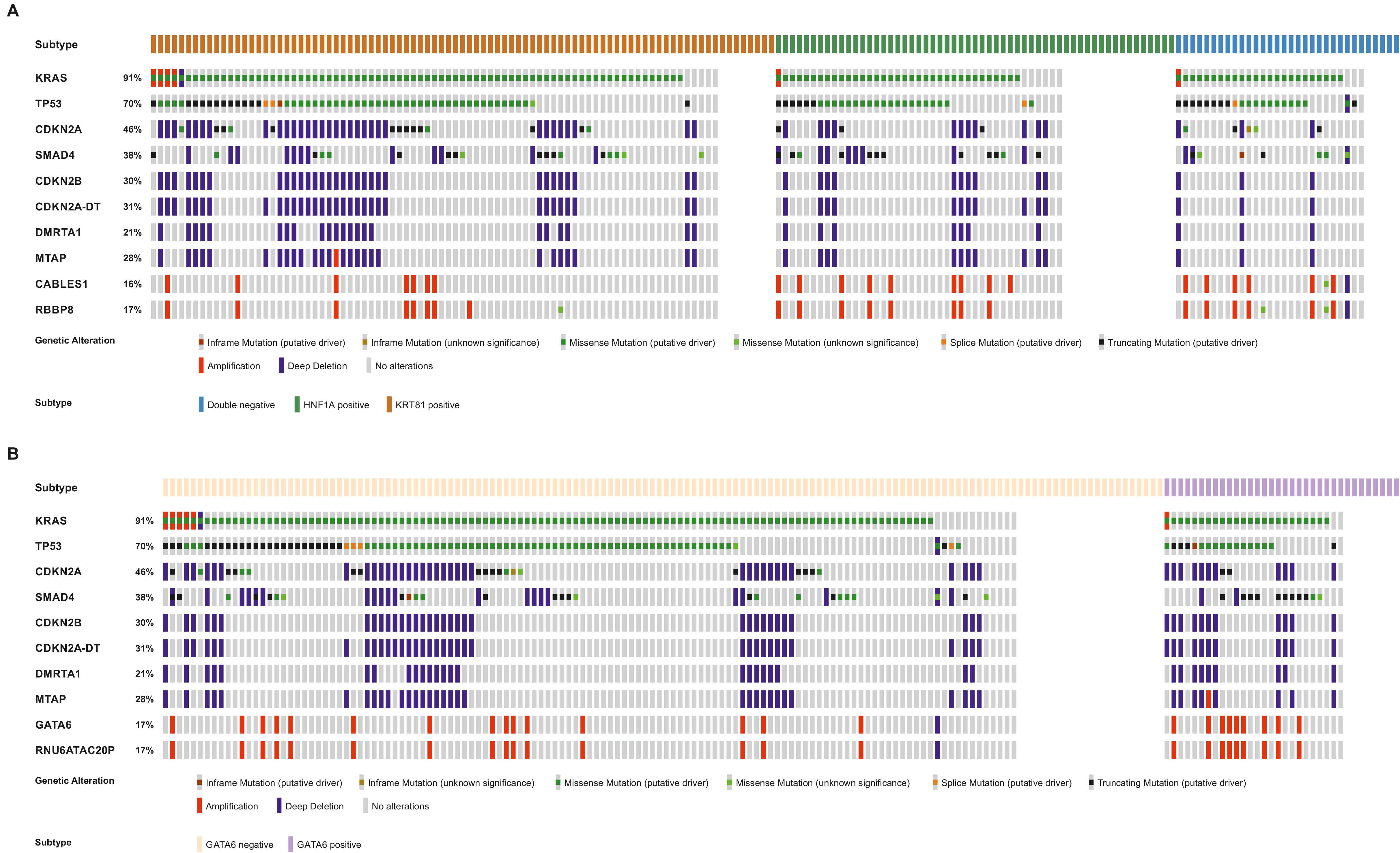

Supplement: Supplementary file 1 [file biomolecules-15-00426-s001.zip › figure_S6.jpg]
